# Patient and surgery related factors associated with fatigue type polyethylene wear on 49 PCA and DURACON retrievals at autopsy and revision

**DOI:** 10.1186/1749-799X-3-8

**Published:** 2008-02-22

**Authors:** Markus Rohrbach, Martin Lüem, Peter E Ochsner

**Affiliations:** 1 Kantonsspital Liestal, Orthopaedic Department, Rheinstrasse 26, 4410 Liestal, Switzerland

## Abstract

**Background:**

Polyethylene wear is an important factor for longevity of total knee arthroplasty. Proven and suspicious factors causing wear can be grouped as material, patient and surgery related. There are more studies correlating design and/or biomaterial factors to in vivo wear than those to patient and surgery related factors. Many retrieval studies just include revision implants and therefore may not be representative. This study is aimed to correlate patient- and surgery- related factors to visual wear score by minimizing design influence and include both autopsy and revision implants. Comparison between the groups was expected to unmask patient and surgery-related factors responsible for wear.

**Methods:**

The amount of joint side wear on polyethylene retrievals was measured using a modification of an established visual wear score. Fatigue type wear was defined as summation of the most severe wear modes of delamination, pitting and cracks. Analysis of patient and surgery related variables suspicious to cause wear included prospectively sampled patient activity which was measured by self reported walking capacity. Statistical analysis was done by univariate analysis of variance. Activity level and implantation time were merged to an index of use and correlated to the wear score.

**Results:**

Wear score after comparable implantation time was significantly less in the autopsy group. Even so, fatigue type wear accounted for 84 and 93 % of total wear score on autopsy and revision implants respectively. A highly significant influence on wear score was found in time of implantation (p = 0.002), level of activity (p = 0.025) and inserts belonging to revision group (p = 0.006). No influence was found for the kind of patella replacement (p = 0.483). Body mass index and accuracy of component alignment had no significant influence on visual wear score. Fatigue-type wear in the medial compartment was closely correlated to the index of use in the autopsy (R^2 ^= 0.383) and the revision group (R^2 ^= 0.813).

**Conclusion:**

The present study's finding of substantial fatigue type wear in both autopsy and revision retrievals supports the theory that polyethylene fatigue strength is generally exceeded in this type of prosthesis. Furthermore, this study correlated fatigue-type polyethylene wear to an index of use as calculated by activity over time. Future retrieval studies may use activity over time as an important patient related factor correlated to the visual wear score. When evaluating total knee arthroplasty routine follow up, the surgeon must think of substantial wear present even without major clinical signs.

## Background

Polyethylene wear in total knee arthroplasty (TKA) is an important limitation to longevity [1,2] because it may cause osteolysis through particle disease [3] or instability due to substantial material loss as previously reported in many posterior cruciate retaining (PCR) designs [4]. Research of polyethylene performance is mostly done by lab studies where influence factors can be controlled more easily. Retrieval analysis has the advantage of reflecting in vivo service, but is done less often due to methodological challenges and reduced component availability. Retrieval studies usually include inlays retrieved at revision [5-8]. However, because they just reflect polyethylene performance from failed arthroplasty, the results may be different from the behaviour of well functioning total joint replacements. There are studies including autopsy retrievals [9,10], but they provide unsatisfactory information on the difference between autopsy and revision retrievals. Also, most studies include a variety of different designs resulting in difficult quantification of non design related influences on the outcome measure.

Generation of polyethylene wear depends upon numerous factors [1,11]. They can be grouped into three basic areas of research interest. Namely polyethylene wear related to patient-, surgery- and hardware- factors. A vast number of studies focus on design and material aspects. Especially researches about polyethylene fabrication and oxidation level due to gamma irradiation are extensive [1,12-15]. On the opposite there are considerably less reports about patient- and surgery related factors. Concerning patient related factors we know about the importance of implantation time, patient weight and age [1,16-18]. Activity level was expected to be a predictor for polyethylene wear in TKA for some time. This was due to technical considerations [19], and the proven fact in total hip arthroplasty [5,8,20] as well as the findings in recent lab studies [21]. To date there is one recent report supporting the hypothesis of increased activity level corresponding to more severe wear in TKA [9]. Surgery-related factors such as tibiofemoral and rotational alignment have been investigated, yet less extensively [18,22,23].

Wear modes can grossly be grouped into adhesive-abrasive and fatigue type wear [24]. The former is represented by polishing and abrasion on visual examination, and the latter by delamination and pitting. Fatigue type wear is generally thought to occur due to repetitive rolling and sliding. This process is thought to be slow, repetitive and eventually exceeds polyethylene fatigue strength as previously reported [24]. It is more serious, because once initiated it can be self perpetuating and soon lead to wear through of the polyethylene [5].

The present study was designed to focus on wear performance in autopsy and revision retrievals and identify patient- and surgery- related factors by minimizing hardware influence. We therefore included retrievals of just one design and manufacturer. Because there are two competing theories regarding the amount of fatigue type wear on autopsy and revision retrievals, our study's first target was to substantiate such a difference and support either theoretical concept. One theory is that inserts from autopsy should have none or minimal fatigue type wear. Assuming that autopsy retrievals had been used with satisfaction and therefore did not have revision. The other theory is that stress concentration in low conforming TKA designs exceeds polyethylene fatigue resistance leading to severe fatigue-type polyethylene wear even in so called well functioning implants, which is supported by lab studies [12,25,26] and other retrieval analysis [25,27].

The second target was to find measurable differences in patient or surgery related factors between autopsy and revision group. Because in theory we expected autopsy retrievals to have lower mean wear score, we also expected patients that used their prosthesis till the end of their life to differ in some of the remaining influencing factors. Additionally for important influencing factors we expected to find a direct correlation to wear score.

## Methods

Between 1994 and 2004 we sampled 49 PCL-retaining primary-TKA implants as part of a program of retrieval analysis at our clinic with special expertise in revision of infected total joint replacement. Reasons for revision were 13 loose components and/or polyethylene wear; 6 infections; 5 knee instabilities and 1 insufficient knee flexion. There were 40 in house patients and 9 referred cases. All in house patients had a routine follow-up with prospective questionnaire, clinical examination and standard x-rays at 1, 2, 5 and 10 years. All implants were made by the same company (Stryker-Howmedica, Allendale). The specimen cohort consisted of 25 inserts from consecutive patients revised at our institution and 24 inserts from autopsy. Table 1 lists the characteristics of the two groups. Values for these items were collected by retrospectively analyzing the patient records. Items are grouped by design, patient and surgery related factors. All inlays were irradiated gamma in air, with the exception of 4 Duracon inlays, which were irradiated in protection gas and subsequently DURATION^® ^stabilized.

**Table 1:** Factors Table

**Independent Variables**	**Autopsy**	**Revision**
**1. Retrieved Inlays [N] **	**24**	**25**
**2. Implantation time [months]**	**104.9 (0.8 to 199.0)**	**92.0 (4.7 to 193.6)**

**Prosthesis Related Factors**		

3. Prosthesis Type [PCA/PCA_Modular_/Duracon]	5/14/5	10/7/8
4. Resin Type [GUR4150/GUR1050]	21/3	21/4
5. Sterilization with N_2 _Protection-Gas	0	4
6. **Patella Replacement [Metal Back/Cemented/Unreplaced]	**4/5/12 (N = 21)**	**10/4/10 (N = 24)**
7. Inlay Thickness According to Manufacturer [mm]	10.4 (7.0 to 21.0)	10.5 (7.0 to 21.0)

**Patient Related Factors**		

8. **Age at implantation [months]	73.6 (53.7 to 87.1)	66.0(48.4 to 80.3)
9. **Walking capacity [Level 1, 2 and 3]	**7/4/6 (N = 17)**	3/8/11**(N = 22)
10. Body Mass Index	23.4 (16.3 to 29.4)	26.6 (16.7 to 39.2)
11. Preoperative Femoro-tibial angle [°]^μ^	0.9 (18 to -28)	1.2 (28 to -18)
12. Knee Pain score at last F-up	0.2 (N = 16)	1.5 (N = 17)
13. Patient Satisfaction score at last F-up	2.6 (N = 16)	1.8 (N = 17)

**Surgery Related Factors**		

14. Postoperative Femoro-tibial angle [°]^μ^	-5.7 (1 to -12)	-3.2 (5 to -12)
15. Tibia component angle frontal plane [°]^μ^	2.4 (5 to -2)	2.3 (6 to -6)
16. Tibia component angle sagittal plane [°]^μ^	1.0 (5 to -9)	-1.9 (2 to -11)
17. Femur component angle frontal plane [°]^μ^	-8.1 (-4 to -13)	-5.8 (12 to -10)
18. Femur component angle sagittal plane [°]^μ^	-4.9 (-5 to 2)	-2.5 (-9 to 7)
19. Index of unacceptable malpositioning	1.1	1.2
20. Patellae with lateralization on axial view	3	10
21. Instability Index	1.0	1.8

Grouped data for all factors entered in cluster analysis. Values for items are given as absolute numbers or mean values with range. Bold items (1, 2, 6, and 9) with most important influence on wear score were analyzed in definitive ANOVA. Remaining differences were tested via separate t-tests. ^μ ^Component alignment angles in frontal and sagittal plane. Negative values indicate varus in the frontal and flexion in the sagittal plane.

**Significant difference between autopsy and revision as estimated by separate t-test (p < 0.05)

**Figure 1:**
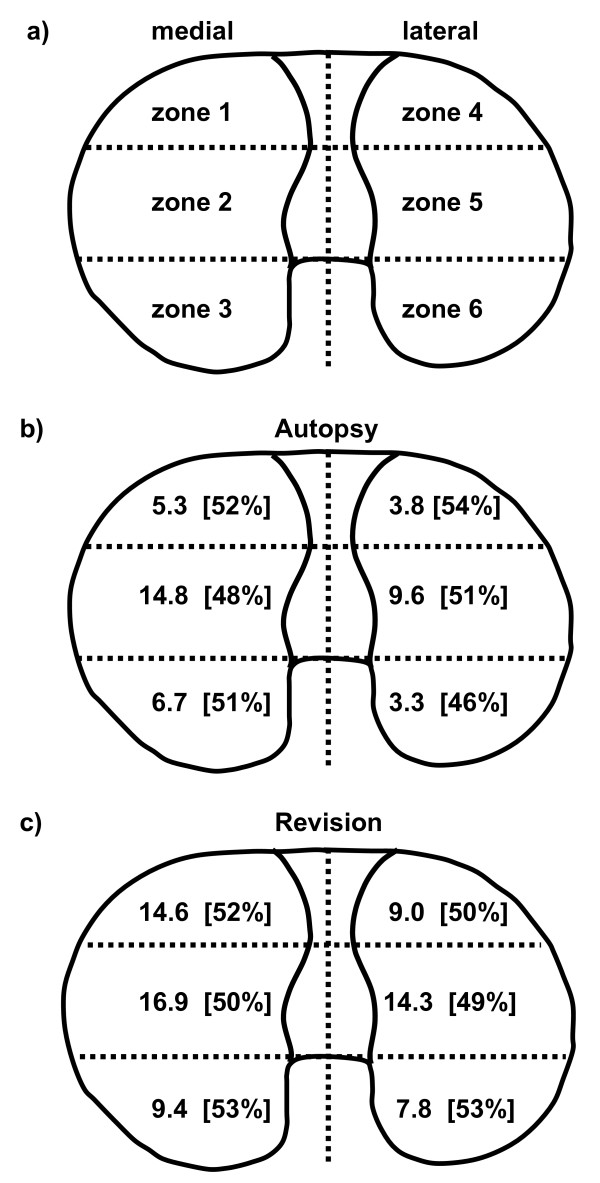
**Zones and scores**. Inlays were divided into six zones with 1–3 always representing medial and 4–6 lateral side (a). Mean total wear score for autopsy (b) and revision retrievals (c). Relative values for fatigue type wear are listed in brackets.

Retrieved polyethylene inserts were photographed and assessed for wear by visual surface examination using a modification of an established wear score from Hood et al. [7,19]. Assessment was done by the second author, who was blinded to all patient-related data. To rule out intra-observer variation, wear rating was done twice several weeks apart. Definitive scores were subsequently calculated as mean values. Each insert's joint side was divided into 6 zones (Fig 1a) in a pattern very similar to that used by Blunn et al [7]. Each zone was rated from 0 (none) to 3 (most severe) for the presence of each of the five wear modes: delamination, cracks, pitting, abrasion and polishing. Delamination was defined as sheets of polyethylene coming off the surface. Cracks were seen in some inlays presenting as white lines at the outer margins going through full thickness. They were graded as 0 (none) to 3 (most severe, with three or more cracks). Pitting was defined as irregularly shaped craters usually 2–3-mm in diameter and 1–2 mm deep. Delamination, pitting and full thickness cracks were defined as fatigue type wear modes. According to most authors they are closely related to stress exceeding material fatigue strength [24,28]. Abrasion was defined as tufted areas resulting from roughening usually when pieces of bone or cement were running over that particular inlay area. This mode was rarely seen and therefore was discarded in the calculation of the total wear score. Polishing was defined as highly polished areas most likely corresponding to adhesive loss of material. Delamination and pitting were the overwhelming majority of wear modes and usually caused substantial loss of material. Thus when calculating the total damage score for each zone, we incorporated a separate factor for loss of material ranging from 0 (none) to 3 (most severe), which was then multiplied with the number for delamination and pitting. For instance if a zone had a severely delaminated polyethylene and therefore gross loss of material the total damage score for delamination was 3 × 3 = 9. The grand total of wear score for one inlay was calculated by summation of scores for the six zones. The theoretical maximum score was 3 × 3 (delamination*material loss) plus 3 × 3 (pitting*material loss) plus 3+3+3 (cracks+pitting+polishing) multiplied by 6 zones = 162. Presence of third bodies (cement and metal particles) and erosion of central peg was noted separately.

### Patient related factors

Patient *activity level *was assessed by using the self reported walking capacity as a rough measure of activity. Prospectively sampled data was available for 30 of the in house patients. In the 9 referred cases *activity level *was assigned according to written preoperative history. Stratification of walking capacity in the questionnaire was simple and expressed as low (0–15 min walking capacity), medium (15–60 min walking capacity) and high (more than 60 min walking capacity). An *index of use *was calculated as the product of numeric *activity level *and implantation time to better reflect the effect of functional demand over time.

Where available the patient's scores for knee pain and satisfaction with the operation were noted. The stratification of these values was similar to that in walking capacity. 0 indicated no pain and no satisfaction, whereas 3 indicated most intensive pain and best satisfaction.

### Surgery related factors

**Figure 2:**
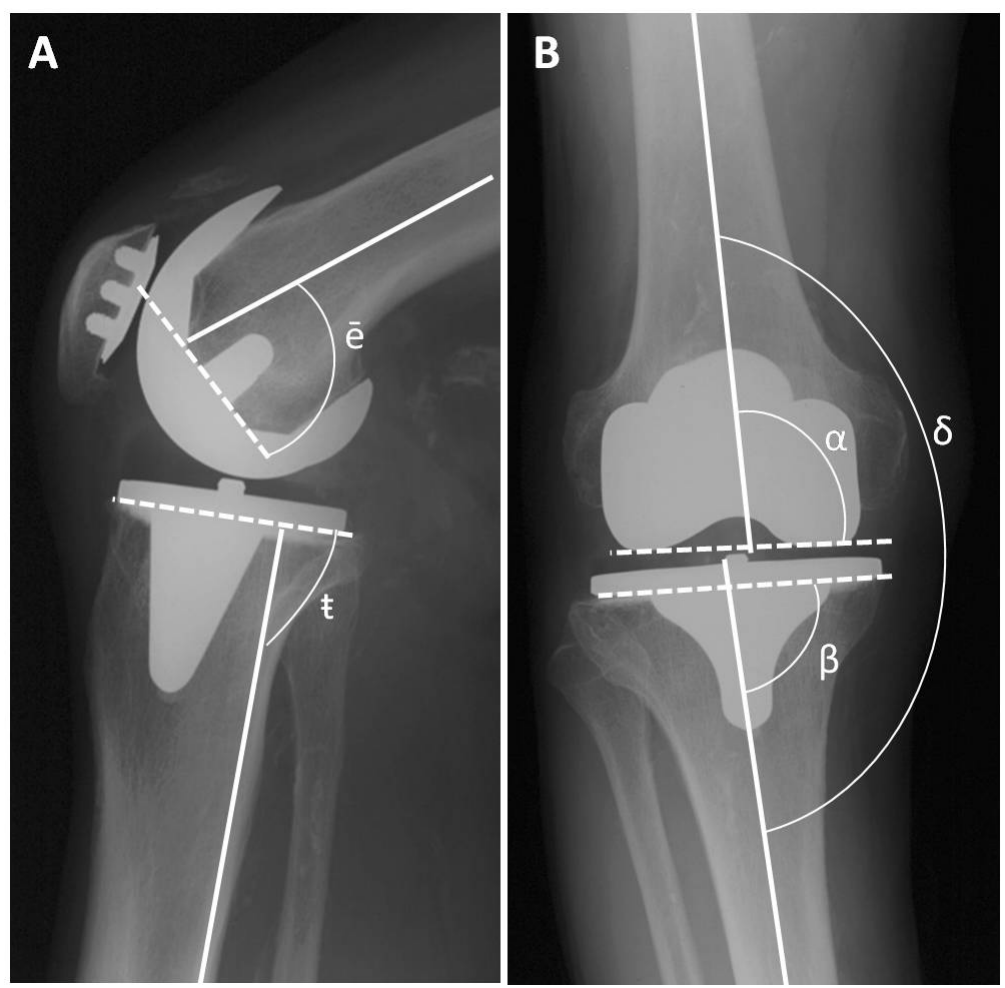
**Surgical accuracy on long leg radiographs**. Component positioning angles were measured on standing long leg radiographs with lateral (A) and antero-posterior-view (B). Angles between component axis (broken line) and mid-tube bone axis (straight line) were measured for tibia component slope (g), femur component flexion-extension (ē), femur component varus-valgus (α) and tibia component varus-valgus (β). Tibiofemoral varus-valgus (δ) was measured between long bone axes. For slope measurements (g), the posterior cortex line served as reference.

Tibiofemoral alignment on long leg radiographs and component positioning angles on both antero-posterior and lateral views were available for measurements in 42 of the cases (Fig 2). An index of unacceptable malalignment was calculated according to x-ray analysis by summation of points. Points were given for tibiofemoral varus-valgus (δ exceeding ± 6°), component positioning in antero-posterior and lateral respect (α, β, g and ē exceeding ± 3°) and patella lateralization on the axial view. In a similar way an index of postoperative instability was calculated according to clinical follow up data. The amount of translation in antero-posterior and sagittal was graded from 1 (normal) to 3 (clearly abnormal) and points were summed to form the index.

### Statistics

To identify influence factors on wear score regression-analysis was done by univariate analysis of variance with wear score being the independent variable and 4 out of the 21 dependent variables listed in Table 1. We attempted to increase statistical power by limiting influence factors entered into definitive analysis, as the number of factors should correspond to the total number of samples divided by 10 in a meaningful regression analysis [13]. Therefore cluster analysis by spearman ranked correlation as similarity measure was done prior to ANOVA. This process yielded the 4 most important factors, namely *implantation time*, *belonging to autopsy or revision group*, *activity level *and *patella replacement*. Separate analysis was done for total wear score as well as medial and lateral compartment wear scores. Also partial wear score for fatigue type wear (delamination, pitting and cracks) was separately analyzed. Retrospective power analysis was computed using alpha = 0.05. Differences between revision and autopsy group for the remaining 17 items in Table 1 were separately evaluated by t-tests.

**Figure 3:**
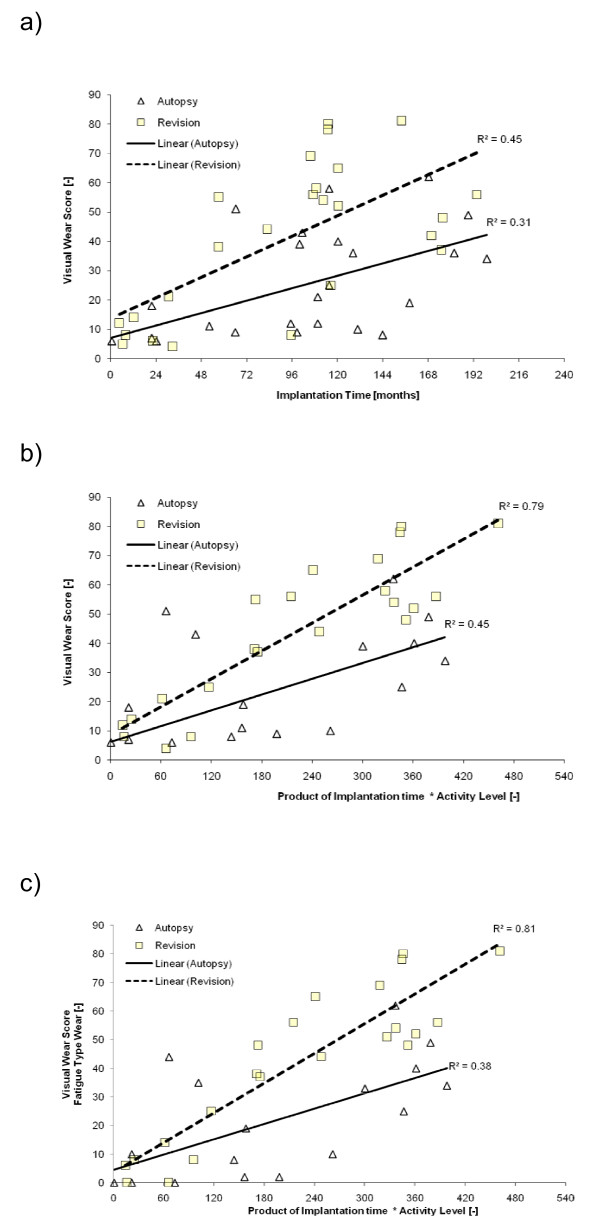
**Wear score vs. Implantation time**. Medial compartment wear score plotted against implantation time (a) and the index of use as calculated by the product of numeric activity level and implantation time in months (b). Partial wear score consisting of fatigue type wear plotted against the index of use (c). R^2 ^in model (b) and (c) is improved compared to model (a) indicating that (b) and (c) are superior in explaining wear score variation.

**Figure 4:**
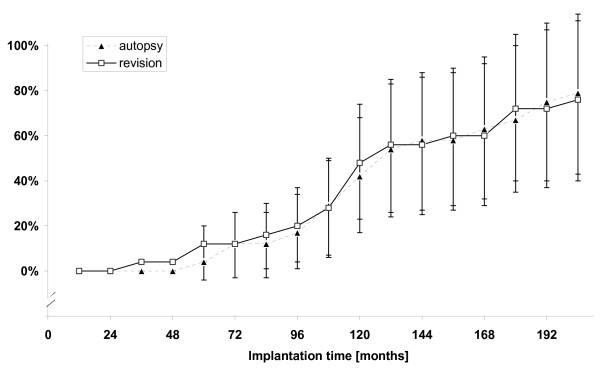
**Probability curves for occurrence of fatigue-type wear**. Probability curves for occurrence of fatigue-type wear with 95% confidence boundaries. Calculation was done via Kaplan-Meier survival estimates and plotted as cumulative hazard plot. Time course for autopsy and revision is not different (Log ranked test ns.) indicating that cumulative risk at a given time point was the same independently from group affiliation.

To test the assumption that inserts with higher wear scores were correlated to more distinctive use, the *index of use *was plotted against total medial (Fig 3b) as well as medial fatigue type wear score (Fig 3c) and linear regression was calculated. Probability curves were drawn to compare cumulative risk of fatigue type wear (delamination, pitting or cracks) and subsequently tested for difference via log ranked test. Calculation was done using the method of Kaplan-Meier and displayed as cumulative hazard plot (Fig 4).

## Results

### Analysis of variance

When analysing total wear score as the dependent variable, observed power was 0.907 for *implantation time*, 0.822 for *belonging to autopsy or revision group*, 0.689 for *activity level *and 0.164 for *patella replacement*. However, in the analysis of medial compartment wear only, observed power for *activity level *was sufficient (0.819), setting the power to the usual limit of 0.8. Univariate ANOVA with the 4 most important factors revealed a highly significant influence on total wear score for *implantation time *(p = 0.002), *activity level *(p = 0.025) and *inserts belonging to revision group *(p = 0.006). No relevant influence was found for *kind of patella replacement *(p = 0.483). These four variables alone were able to predict 53.6% of the total variation in the dependent variable. The same calculation was done for medial and lateral compartments separately. P-values were computed for *implantation time *(0.001/0.021), *activity level *(0.009/0.167), *inserts belonging to revision group *(0.016/0.008) and *kind of patella replacement *(0.43/0.67) corresponding to medial and lateral compartments respectively.

### Comparison between autopsy and revision

**Figure 5:**
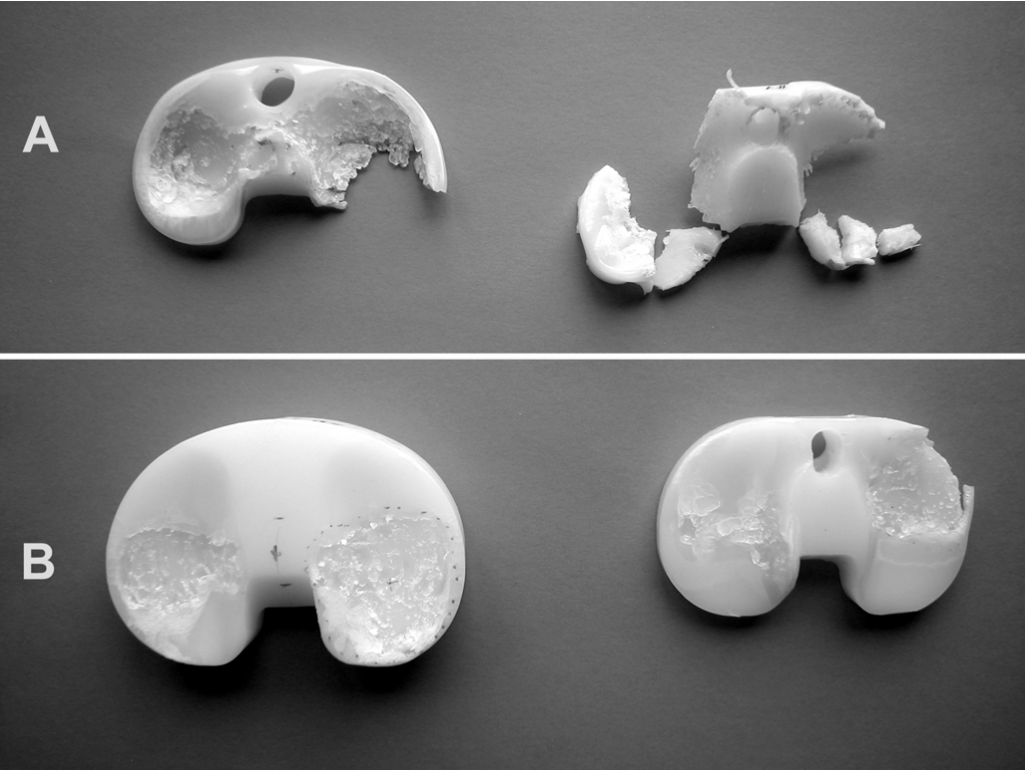
**The two cases with highest wear score**. The two cases with highest damage score for each group; revision (A) and autopsy (B).

Total visual wear score was significantly lower for inserts from autopsy compared to revision as demonstrated by ANOVA (p = 0.006). The same was true for all comparative wear scores in the six zones (Fig 1b, c). Yet the relative amount of fatigue type wear was high in both groups. Fatigue type wear accounted for 84 % and 93 % of total wear score on autopsy and revision implants respectively. The same was true for relative values of fatigue type wear in the six partial zones (Fig 1b, c). This finding is illustrated by the two inlays with highest wear scores from both groups that mainly differ in the amount, but not the type of wear (Fig 5).

The groups were very similar in terms of most influence factors, except for the following significant differences (Tab. 1). Retrievals from revision were implanted earlier in life and originated from more active patients. There was a mean under-correction of postoperative valgus in the revision group (3.2° valgus) compared to a sufficient mean correction in the autopsy group (5.7° valgus). There was more prominent wear score in the medial compared to the lateral compartment as estimated by a separate t-test for correlated samples (p < 0.001). Individuals requiring revision were significantly less satisfied and had more pain at the last follow up.

Visual wear scores in both autopsy and revision increased linearly with length of implantation time. This finding was uniform for total wear score as well as medial and lateral sub scores. Since observed statistical power was good for medial wear score, this sub score is graphically depicted (Fig 3a–c). R^2 ^in model (a) was 0.31 and 0.45 for autopsy and revision respectively.

Probability curves for occurrence of fatigue-type wear in autopsy and revision group were not statistically different (Fig 4).

### Patient related factors

Activity was an important influence on wear score as presented above. To test the assumption that inserts with higher wear scores were correlated to more distinctive use, the *index of use *was plotted against wear score (Fig 3b). This resulted in improved R^2 ^(0.45 and 0.79) indicating that a model incorporating *activity level *is more accurate in predicting wear score than the model with *implantation time *alone. The highest R^2 ^was observed when plotting fatigue type wear against the *index of use *in the revision group (0.81). Age at implantation, body mass index, preoperative tibiofemoral alignment and postoperative pain-/satisfaction scores had no significant influence on visual wear score. Age was not correlated to activity level (p = 0.603).

### Surgery related factors

There was no correlation of postoperative tibiofemoral alignment to wear score. The same held true for all component positioning angles measured in two planes, the index of unacceptable malalignment and the index of instability. There was an increased fraction of lateralized patella in the revision group 10/25 compared to the autopsy group 3/24 (Tab. 1).

## Discussion

### Comparison between autopsy and revision

The first question addressed in the present study was the comparison between the amount of fatigue type wear at both ultimate endpoints revision and autopsy. Because of the high stress load in low conforming designs, one of the theories was to find some fatigue related wear even on inserts that lasted till the end of life. Consequently th**e **finding of fatigue type wear on autopsy retrievals was not a surprise, yet the extent of delamination, pitting and cracks seen was astonishingly high. Also there was equal relative contribution of fatigue type wear to total wear score in both groups. We interpret these data to support the hypothesis that inherent polyethylene overloads due to design and material issues in this type of prosthesis. As a number of previous studies have already reported [16,29,30], we found fatigue type wear on almost all investigated inlays whether they needed revision or not. Further support for the theory that both groups underwent a similar time course to polyethylene fatigue failure, can be derived from probability curves for occurrence of fatigue-type wear (Fig 4). There was no difference in cumulative hazard plots for occurrence of fatigue type wear between autopsy and revision. Interestingly there is close correspondence of this prediction to the failure rate of 11% at 8 years reported in a large PCA follow-up study [30]. All this stands in line with findings from in vitro studies, which calculated stress loads exceeding material properties in similar TKA designs [12,25,31]. While cumulative risk of fatigue type wear was the same at a given time point in both groups, the revision group had an increased time rate in wear score compared to the autopsy group (Fig 3a–c). This is plausible since it is known that fatigue type wear once initiated is self perpetuating and may continue at an increased time course [5]. We interpret increased time rate in revision group as an indication that individuals in the revision group feature additional factors to accelerate wear rate beyond the time where fatigue failure occurred. We propose increased patient activity in the revision group to be a main factor.

Our study's second question focused on patient and surgery related influences on wear score. In this study particular care was taken to minimize the influence of material properties and design by restricting inclusion to only one manufacturer and one design. Consequently differences in wear score should reflect mainly a difference in patient and surgery related variables. Patient and surgery related influences are discussed separately.

### Patient related factors

There was a highly significant influence of implantation time on wear score as calculated by ANOVA. In that respect our data are in close accordance with many previous studies in total knee and total hip arthroplasty [5,7-9,19,27,32,33]. When total wear score was plotted against the index of use (product of activity level and implantation time), regression coefficient was improved compared to the model with implantation time alone (Fig 3a, b). It should be noted that fatigue type damage (delamination, pitting and cracks) had an even closer correlation to the index of use (Fig 3c) than to total wear score (Fig 3b). We conclude polyethylene wear to be rather a function of use (activity over time) than activity or implantation time alone. More precisely, it is mainly fatigue type wear (delamination, pitting and cracks) that correlate**s **to increasing use. Activity, as a cause for polyethylene wear and a risk for revision surgery, is still discussed contradictory in recent literature. A recent report suggested that activity over time was a very important factor causing wear [34], on the other hand increased activity is not necessarily associated with an increased risk for revision [17]. To date, there is only few data from retrieval analysis to support either hypothesis. Only one retrieval study was able to provide retrieval data supporting the theory that activity level was a factor adding to the destructive course of polyethylene [9]. However, they assigned activity level retrospectively and the outcome measure was polyethylene deformation and creep, which is different from fatigue type polyethylene wear (delamination, pitting and cracks). In this context, our findings may be regarded as unique.

We didn't find body mass index to have an effect on visual wear score, which is in line with previous retrieval studies [5,10,35,36]. This may partially be explained by the fact that obese patients are more likely to have decreased activity which counteracts the wear generating effect of increased weight [37]. Additionally wear generation must not be seen in the light of contact pressure only, but rather a system of both contact load and mechanics as described later.

### Surgery related factors

There was a significant overall postoperative under-correction of tibiofemoral valgus in the revision group (mean 3.2° valgus) compared to a sufficient correction in the autopsy group (mean 5.7° valgus). There was also significantly increased wear on the medial side (p < 0.001). Partial explanation for the incidence of increased medial wear score may be the fact that even correctly aligned knees experience increased load transmission through the medial side [38,39]. However, we didn't find correlation between increasing varus and medial wear score. One would expect increasing compartment pressure due to malalignment to cause more wear and thus postulate correlation of increasing varus to medial wear and increasing valgus to lateral wear. However, there was no correlation of tibiofemoral alignment to wear score. The same was true for all component positioning angles correlated to visual wear score. This finding is in accordance with other PCA-retrieval studies [29], yet it may be somewhat surprising to a biomechanical mindset. Therefore we emphasize that though polyethylene wear is contact load dependent [40-42] it has also been shown that wear generation is a function of total sliding distance in the first place [24,41,43]. Sliding distance at the tibiofemoral junction obviously correlates to patient activity. So we conclude that in our study population, wear generating effects due to increased *functional demand *were more important than increased *contact load *as described by patient weight or tibiofemoral alignment.

Even though in our study there was only no significant association of surgery related variables to wear score, we like to point out that raised contact load due to surgical performance is not negligible at all. Several previous reports illustrate the critical role of surgical technique in generating eccentric loads [44] and increased compartment pressure [31,45-47] which can cause catastrophic failure [22,48]. We were not able to demonstrate correlation of tibiofemoral instability to wear score as previous studies have done [7]. However, we do not encourage surgeons to slowdown improving stable knee mechanics through ligament balancing, since it has been shown that multidirectional traction as present in instable knees can lead to elevated wear rates [49]. Last but not least there was an elevated fraction of lateralized patella in the revision group. This should be regarded as an indication to avoid lateral tracking patella.

### Limitations

Despite our aim to limit the influence of design, we were not able to formally rule out a possible influence of different patella replacements. In the beginning the old PCA was implanted with metal backed patella implants. Previous studies have found metal backed patella to perform worse because of third body wear. In the present study, ANOVA showed that the kind of patella replacement was not an important influence factor.

Activity level was measured indirectly by self reported maximal walking capacity. This is a rough measure and validity is a potential issue, because there is no guarantee that a patient really uses his capacity to the full extent. We emphasize that self reported activity has been used before [9] and there are reports about correlation of self reported and objectively measured activity levels [50]. Even though our stratification resembles to that of the University of California Los Angeles activity score, it has not been tested against pedometer measurement. However, our simple activity measure via walking capacity yielded an important factor in statistical analysis. It should be noted that our measure of activity was not correlated to age, which stands in line with previous reports [51].

Generally visual rating systems are mostly based on measuring an area affected by wear and not on changes of friction coefficient [52] or polyethylene debris generation. Therefore such rating systems may not be able to detect destruction before visible polyethylene changes or ongoing fatigue type wear after its first occurrence. Though we included a factor for material loss in our rating system, this may not have been accurate enough. Theoretically we may have missed subtle changes and therefore were not able to find further variables correlated to wear.

Sample size was moderate and yielded in need to reduce variables for ANOVA. We cannot be sure to have detected all possible influences of variables excluded after cluster analysis. Similarly statistical power for activity level as a major influence did not reach the 80% level in ANOVA with total wear score as dependent variable. However, for medial compartment wear statistical power was sufficient. We propose that further retrieval investigation would be needed to clarify influence of potentially undetected factors.

Despite these limitations, we believe that retrieval studies with long-term follow up and specimen from revision and autopsy are a necessity to gather appropriate performance data. Newer designs and inlays with improved in-vitro performance [53] should be investigated with records of activity level.

## Conclusion

**1) Comparison autopsy and revision**: The present study's finding of substantial fatigue type wear in both autopsy and revision retrievals supports the theory that polyethylene fatigue strength is generally exceeded in this type of prosthesis

**2) Patient related factors**: Fatigue type wear in this type of prosthesis is closely related to the index of use as calculated by activity over time. We conclude that wear is promoted by activity over time. The index of use may be helpful for future investigation.

**3) Surgery related factors**: None of the alignment variables could be correlated to visual wear score. We conclude that with respect to visual wear score the effects of increased *functional demand *were more important than increased *contact load *as described by tibiofemoral alignment.

## Competing interests

The authors declare no competing interests.

## Authors' contributions

The following authors have designed the study: MR, ML and PEO gathered the data. MR wrote the initial drafts. ML ensured the accuracy of the visual wear, score and analysis.
